# Comparison of breast sequential and simultaneous integrated boost using the biologically effective dose volume histogram (BEDVH)

**DOI:** 10.1186/s13014-016-0590-1

**Published:** 2016-02-02

**Authors:** Moamen M. O. M. Aly, Yasser Abo-Madyan, Lennart Jahnke, Frederik Wenz, Gerhard Glatting

**Affiliations:** Medical Radiation Physics/Radiation Protection, Universitätsmedizin Mannheim, Medical Faculty Mannheim, Heidelberg University, Mannheim, Germany; Department of Radiotherapy and Nuclear Medicine, South Egypt Cancer Institute, Assiut University, Assiut, Egypt; Department of Radiation Oncology, Universitätsmedizin Mannheim, Medical Faculty Mannheim, Heidelberg University, Mannheim, Germany; Department of Radiation Oncology and Nuclear Medicine (NEMROCK), Faculty of Medicine, Cairo University, Cairo, Egypt

**Keywords:** Breast cancer radiotherapy, Sequential boost (SEQ), Simultaneous integrated boost (SIB), Biologically effective dose (BED), Biologically effective dose volume histogram (BEDVH)

## Abstract

**Purpose:**

A method is presented to radiobiologically compare sequential (SEQ) and simultaneously integrated boost (SIB) breast radiotherapy.

**Methods:**

The method is based on identically prescribed biologically effective dose (iso-BED) which was achieved by different prescribed doses due to different fractionation schemes. It is performed by converting the calculated three-dimensional dose distribution to the corresponding BED distribution taking into consideration the different number of fractions for generic α/β ratios. A cumulative BED volume histogram (BEDVH) is then derived from the BED distribution and is compared for the two delivery schemes. Ten breast cancer patients (4 right-sided and 6 left-sided) were investigated. Two tangential intensity modulated whole breast beams with two other oblique (with different gantry angles) beams for the boost volume were used. The boost and the breast target volumes with either α/β = 10 or 3 Gy, and ipsi-lateral and contra-lateral lungs, heart, and contra-lateral breast as organs at risk (OARs) with α/β = 3 Gy were compared.

**Results:**

Based on the BEDVH comparisons, the use of SIB reduced the biological breast mean dose by about 3 %, the ipsi-lateral lung and heart by about 10 %, and contra-lateral breast and lung by about 7 %.

**Conclusion:**

BED based comparisons should always be used in comparing plans that have different fraction sizes. SIB schemes are dosimetrically more advantageous than SEQ in breast target volume and OARs for equal prescribed BEDs for breast and boost.

## Background

Adjuvant radiotherapy following breast conserving surgery is still usually performed by homogenous irradiation of the whole breast using doses of 1.8–2 Gy per fraction up to a total dose of about 50 Gy, although hypofractionated regimens are being used more and more often and were shown to be well tolerated [1]. Frequently, a sequential boost (SEQ) to the tumor bed follows, as it is known to be the area of highest subclinical tumor cell contamination [2]. Recently, many authors suggested the use of simultaneously integrated boost (SIB) using doses of 2.3–2.4 Gy to the tumor bed as it showed to be dosimetrically better, more convenient due to the shorter treatment time and well tolerated [3, 4]. Moreover, hypofractionation with SIB has been evaluated in a few trials and appears to be feasible with no severe adverse events [1, 3–6].

Starting from the linear-quadratic (LQ) model of cell survival, Barendsen [7] introduced the extrapolated response dose, which was later termed the biologically effective dose (BED) [8]. The BED concept has been widely used in radiotherapy for conversion between different fractionation schemes [8–10] and has become the clinical reference tool to estimate the malignant and normal biological effects in tissues [11]. An alpha to beta ratio (α/β) of 10 Gy for tumor response and α/β of 3 Gy for late-responding normal tissues were used to determine the SIB prescribed BED for the breast and the boost volumes from the traditionally used SEQ prescribed BED [12]. Recent reports suggest that healthy breast tissues as well as the tumor are sensitive to fraction size with an α/β of 5 Gy or less [13–15].

It is not an easy task to compare and assess two dose distributions in terms of tumor control (TCP) and normal tissue complication (NTCP) probabilities, especially when having different fractionation schedules. Many of these comparisons are made by using dose volume histograms (DVHs) [16, 17]. Different studies have shown a disparity between the physical and biological dose distribution [18, 19]. The biological effects do not depend simply on the distribution of physical dose, but are a non-linear function of the number and the size (dose) of fractions [18, 19]. Therefore, using DVHs to compare or evaluate two plans with different prescribed doses and number of fractions could be misleading, as it does not adequately represent the biological effect, even when comparing two plans with the same prescribed biologically effective dose. The use of SIB techniques further complicates the evaluation procedure, and puts an emphasis on the need to analyze the biological effectiveness of the nominal dose based on the number of given fractions. More recent studies suggest the use of LQ model to interpret the DVH [18] and even to reduce the DVH to a single biological parameter, such as equivalent uniform BED (EUBED) [20]. A method that incorporates all biological parameters is demanded to radiobiologically compare different treatment courses.

Here we present a novel method to rigorously compare the biological effective doses of sequential and simultaneous integrated boost for breast cancer not only for the prescribed BED but also for the 3D BED distribution for target volumes and organs at risk (OARs) taking in consideration different α/β values and number of fractions.

## Materials and methods

### Patient selection and image data

Ten female breast cancer patients (4 right-sided and 6 left-sided) treated in the Department of Radiation Oncology, University Medical Center Mannheim/Germany were retrospectively selected. Selection criteria were average breast size with well-located tumor bed. The planning computed tomography (CT) data-sets were acquired with 5 mm slice thickness in supine position with the use of a wing board for arm positioning above the head by a CT-simulator (Brilliance CT Big Bore, Philips, Cleveland, OH, USA).

Breast volumes include the total glandular breast tissue cropped 4 mm inside the skin contour (the affected side and the contra-lateral breast (CBreast)), the ipsi-lateral lung (ILung), contra-lateral lung (CLung), and heart were delineated. The tumor-bed was delineated by an experienced radiation oncologist according to the scar, pre- and post-operative radiological changes within the breast tissue, the surgical report and/or the presence of surgical clips. A setup safety margin of 5 mm was automatically added to this tumor-bed to create the boost planning target volume (PTV_boost_). This safety margin was constrained to 5 mm under the skin contour. The affected breast volume was considered the whole breast planning target volume (PTV_breast_).

### Biologically effective dose (BED)

The concept of biologically effective dose (BED) is commonly used for iso-effective dose fractionation calculation [9]. It is derived from the LQ model and is defined as:

1 $$ BED = nd\left[1 + \frac{d}{\alpha /\beta}\right] $$

where *n* is the number of fractions, *d* is the dose per fraction, and *α/β* is the ratio of the radiosensitivity coefficients.

Using Eq. (1), the prescribed dose per fraction (*d*_*2*_) for a different number of fractions *n*_*2*_ that is biologically iso-effective can be calculated according to

2 $$ {d}_2 = \sqrt{{\left(\frac{\alpha /\beta }{2}\right)}^2 + \frac{\alpha /\beta \times BED}{n_2}} - \left(\frac{\alpha /\beta }{2}\right) $$

where *BED* is the chosen identically prescribed biologically effective dose (iso-BED).

### Treatment planning and prescriptions

For each patient, a sequential boost (SEQ) and two simultaneously integrated boost (SIB) intensity modulated radiotherapy (IMRT) plans were generated using a Monaco treatment planning system (v3.3, Elekta AB, Stockholm, Sweden).

The SEQ plans were composed of two different plans that were optimized separately. The first plan was a whole breast plan which consisted of two tangential IMRT beams (medial and lateral tangents) assigned to the PTV_breast_ with a prescription dose of 50 Gy in 25 fractions (BED = 60.0 Gy_10_ and 83.3 Gy_3_). The second plan was the boost plan which consisted of two coplanar IMRT oblique beams assigned to the PTV_boost_ with individually selected gantry angles to prevent any unnecessary dose to OARs especially the ipsi-lateral lung and contralateral breast. The prescribed dose for the boost plan was 16 Gy to the PTV_boost_ in 8 fractions (BED = 19.2 Gy_10_ and 26.7 Gy_3_) [21].

The SIB plans were achieved by combining the previously selected tangential beams assigned to the PTV_breast_ and the two coplanar oblique beams assigned to the PTV_boost_ in a single optimized plan, i.e., the gantry angles were the same for each patient as in the SEQ. The first SIB plan (SIB_10_) was for prescribed doses of 2.3 Gy to the PTV_boost_ and 1.8 Gy to the PTV_breast_ in 28 fractions, which correspond to total doses of 64.4 Gy and 50.4 Gy, respectively. The second SIB plan (SIB_3_) was optimized for prescribed doses of 2.25 Gy to the PTV_boost_ and 1.84 Gy to the PTV_breast_ in 28 fractions, which correspond to total doses of 62.9 Gy and 51.5 Gy, respectively.

The optimization prescription aimed to deliver 95 % of the prescribed dose to at least 95 % of the target volumes and to minimize the volume receiving > 107 % of the boost dose. Having reached these criteria, additional effort was made to reduce dose to OARs individually for each patient starting from the proper choice of gantry angles to the fine-tuning of the prescription cost functions. All plans were normalized to a median PTV_boost_ dose equal to the prescribed dose.

### BED and BED-volume histogram (BEDVH)

The 3D dose distribution matrices of the SEQ plan (the whole breast plan and the boost plan, separately) and the two SIB plans for each patient were exported as DICOM files. DICOM files were manipulated using Computational Environment for Radiotherapy Research (CERR) software [22]. The BED calculations were performed using a code written in MATLAB (R2013a, The MathWorks, Natick, MA). Each voxel dose was converted to the corresponding BED using equation (1) and taking into account the number of fractions and the different α/β values for tumor and OARs. For all OARs, α/β = 3 Gy was used in all plans. For PTV_breast_ and PTV_boost_ in SEQ plans, α/β = 10 Gy and 3 Gy were used to calculate the BED (BED_10_ and BED_3_); while in SIB_10_ and SIB_3_ plans BED_10_ and BED_3_ were calculated, respectively.

Having converted the 3D dose matrices to 3D BED matrices, cumulative BED volume histograms (BEDVH) were generated and compared.

### Statistical analysis

Descriptive statistics of the data are presented as mean ± standard deviation (SD). The differences of the mean BEDs between the two schemes were compared and analyzed using the two-tailed paired t test or the Wilcoxon matched paired test using GraphPad Prism version 6.04 for Windows (GraphPad Software, La Jolla California USA, www.graphpad.com). Statistically significant differences were assumed for a significance level of *p* <0.05.

## Results

**Fig. 1:**
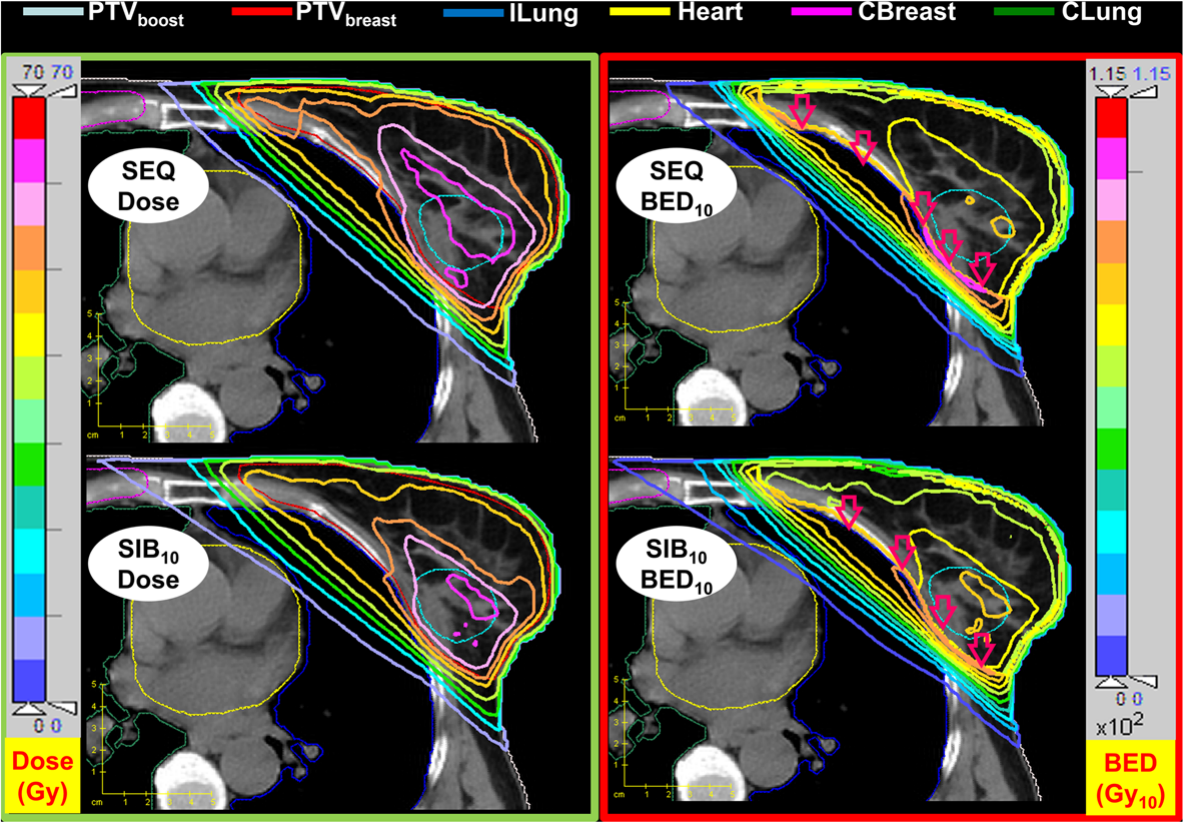
Dose (*left column* and BED (*right column*) distribution for a representative case using the sequential boost and the simultaneously integrated boost schemes employing the same prescribed biologically effective dose with α/β = 3 Gy for all OARs and α/β = 10 Gy for target volumes in 28 fractions. Due to the difference in α/β, the maximum BED values occur outside the target volumes (*red arrows*)

**Fig. 2:**
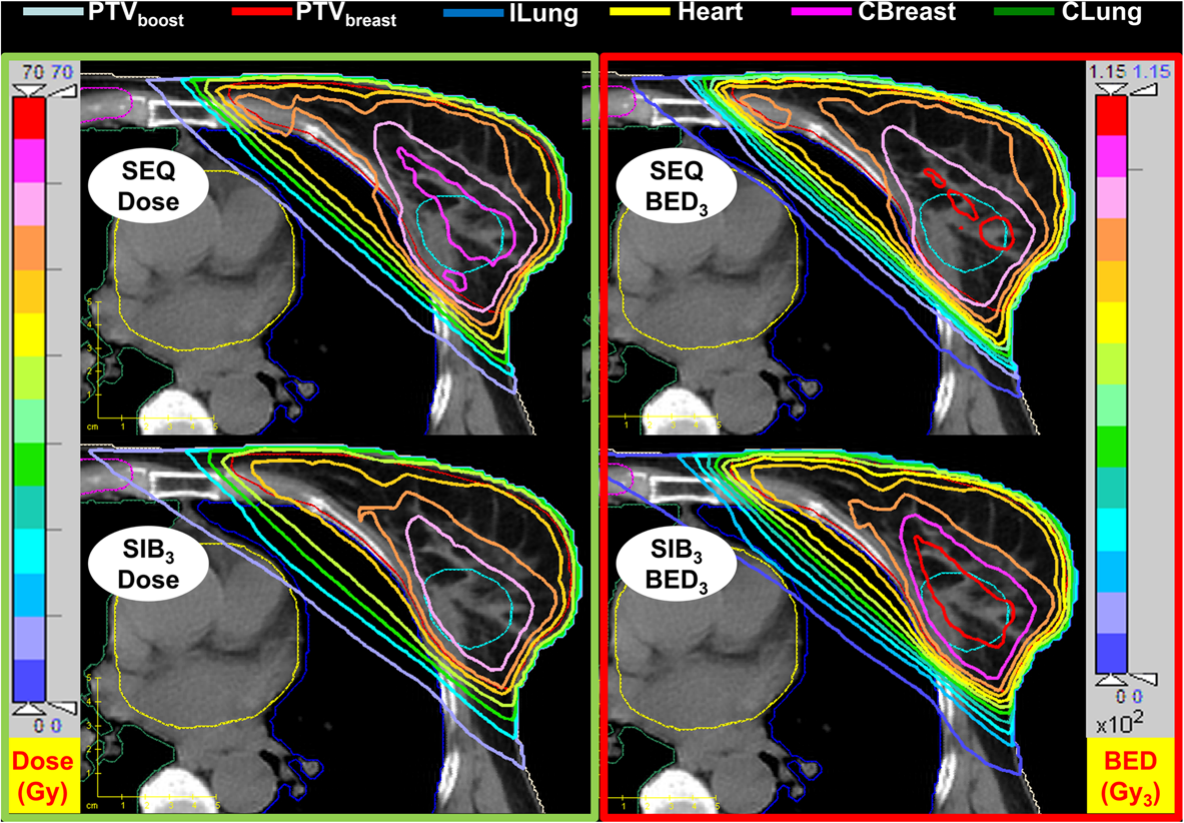
Dose (*left column*) and BED (*right column*) distribution for a representative case using the sequential boost plan and the simultaneously integrated boost plans employing the same prescribed biologically effective dose with α/β = 3 Gy for all OARs and target volumes in 28 fractions. The PTV_boost_ BED target coverage in the SIB plan is better than that of the SEQ plan (compare also Table 2)

**Fig. 3:**
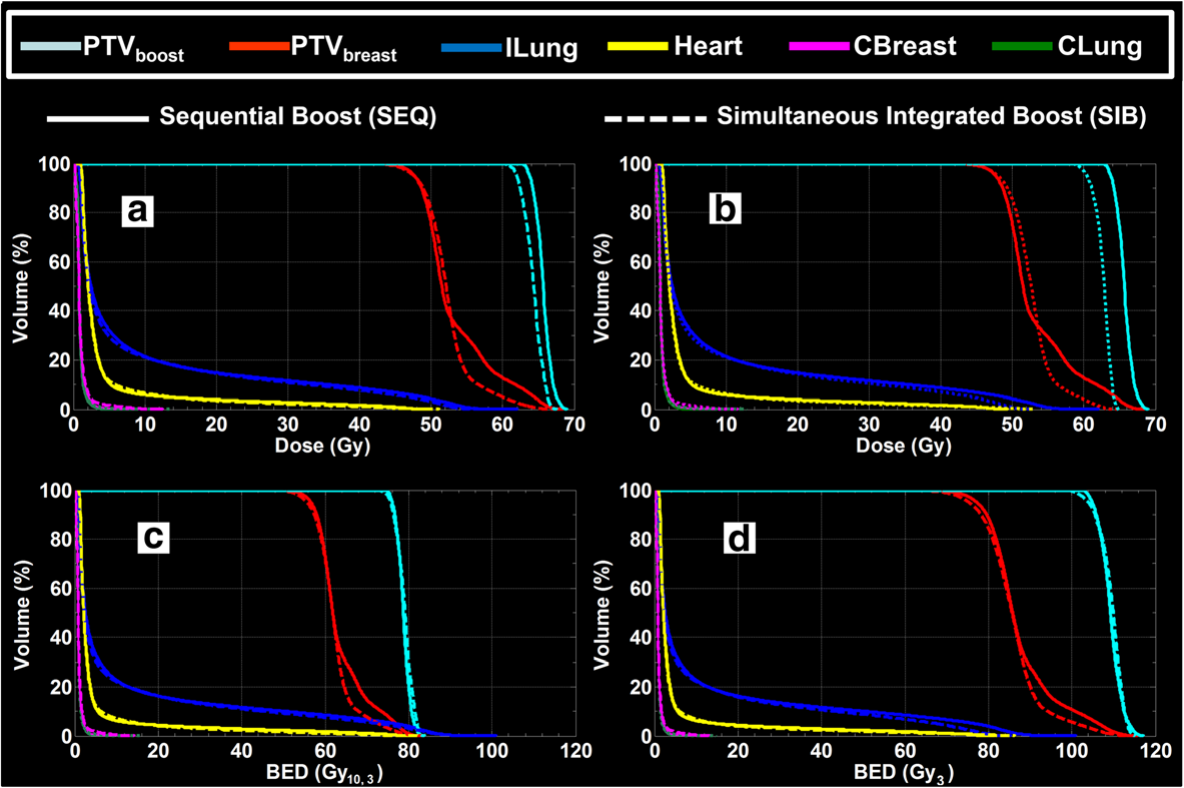
Cumulative dose (**a** and **b**) and BED (**c** and **d**) volume histograms of the representative case using sequential boost (*solid line*) and the corresponding simultaneously integrated boost (*dotted line*) plans. The BED was calculated using the same prescribed biologically effective dose with α/β = 3 Gy for OARs and α/β = 10 Gy (**a** and **c**) and 3 Gy (**b** and **d**) for boost and breast target volumes

The PTV_boost_ and PTV_breast_ volumes were (47 ± 27) cm^3^ and (1124 ± 435) cm^3^, respectively. Table 1 presents the beams angles and the PTV_boost_ locations. Figures 1 and 2 show a CT image of a representative case with the dose and the BED distribution of the sequential boost plan and the simultaneous integrated boost plans. The BED distributions were calculated using α/β = 3 Gy for all OARs and either α/β = 10 Gy (in Fig. 1) or 3 Gy (in Fig. 2) for target volumes in 28 fractions. The figures demonstrate how the BED differs from the dose distribution. In Fig. 1, and due to the difference in α/β between target volumes and OAR, the maximum BED values can be seen outside the target volumes concentrated in the lung and the rest of chest wall (red arrows). The figures also demonstrate the advantages of the SIB delivery scheme over the SEQ due to the improved homogeneity in both dose and BED distribution. This difference is due to the different prescribed doses and because in SEQ plans the whole-breast and the boost plans were optimized separately and then combined, while in the SIB plans the four fields (two tangents for the whole breast and two coplanar for boost) were optimized simultaneously within a single plan.

**Table 1:** Summary of beam angles used in both the sequential boost (SEQ) and the simultaneous integrated boost (SIB) schemes for the ten studied patients (mean ± SD)

Angle (°)	Medial Tang	Medial Boost	Lateral Boost	Lateral Tang
Left-sided ^a^	308 ± 3	348 ± 3	111 ± 8	131 ± 3
Right-sided ^b^	53 ± 4	21 ± 11	276 ± 21	229 ± 4

^a^Group of 6 patients with left-sided breast tumor. PTV_boost_ locations were upper/outer quadrate, central quadrate, and lower/outer quadrate in three, two, and one patient respectively

^b^Group of 4 patients with right-sided breast tumor. PTV_boost_ locations were upper/outer quadrate, lower/outer quadrate, and upper/inner quadrate in two, one, and one patient respectively

Figure 3 presents the cumulative dose and BED volume histograms of the sequential boost plan in comparison to the simultaneously integrated boost plans using the same prescribed biologically effective dose with α/β = 3 Gy for all OARs and either α/β = 10 Gy or 3 Gy for boost and breast target volumes in 28 fraction for the representative case. The figure demonstrates the advantage of SIB plans over the SEQ plans due to the reduction of the over-dose outside the PTV_boost_ that reduces the PTV_breast_ hot-spot and ipsi-lateral organs mean doses. The figure shows also that the BED is not a simple transformation of the structure’s dose where different (depending on the α/β) non-linear scaling for the structure’s doses can be seen for the two target volumes.

Table 2 gives the comparison of the mean dose and BED between the sequential boost plans and the simultaneous integrated boost plans for all structures of the ten studied patients. The absolute and relative differences in the mean dose and BED between the two delivery schemes for all structures of the ten studied patients are presented in Table 3. Both, PTV_boost_ and PTV_breast_ achieved a lower deviation from the prescribed BED in the SIB cases (Table 2), reflecting improved performance of optimization in one step compared to optimization in two steps for the SEQ cases. The most relevant BED_3_ values for PTV_breast_, ILung and Heart_left_ were significantly (*p* <0.05) reduced in average by 2, 11 and 8 %, respectively, thus demonstrating a better sparing of OARs for the same, i.e., non-significantly different, BED in the boost target volume. For the other OARs (Table 3) also a reduction is seen, which however is not significant. This could possibly be attributed to their larger distance to the PTV_boost_ and thus a smaller effect of the reduced dose of the SIB plans. However, larger patient groups would probably render also this reduction as significant as less total dose is applied to the PTVs (Table 3).

**Table 2:** Comparison of mean dose and biologically effective dose (using either α/β = 10 Gy (BED_10_) or 3 Gy (BED_3_) for tumor volumes and α/β = 3 Gy for all OARs) between sequential boost (SEQ) and the simultaneous integrated boost (SIB) schemes for all structures of the ten studied patients (mean ± SD)

	SEQ			SIB_10_		SIB_3_	
	Dose (Gy)	BED_10_ (Gy_10_)	BED_3_ (Gy_3_)	Dose (Gy)	BED_10_ (Gy_10_)	Dose	BED_3_ (Gy_3_)
PTV_boost_ Prescription	66.0	79.2	110.0	64.4	79.2	62.9	110.0
PTV_breast_ Prescription	50.0	60.0	83.3	50.4	60.0	51.5	83.3
PTV_boost_	65.7 ± 0.7	78.7 ± 1.0	109.2 ± 1.7	64.2 ± 0.1	78.9 ± 0.1	62.6 ± 0.1	109.4 ± 0.3
PTV_breast_	53.4 ± 0.7	63.8 ± 0.8	88.0 ± 1.0	52.2 ± 0.4	61.9 ± 0.6	52.8 ± 0.5	86.2 ± 1.0
ILung	9.0 ± 1.8	12.2 ± 2.7		8.2 ± 1.7	11.0 ± 2.5	8.2 ± 1.6	10.9 ± 2.4
CBreast	1.2 ± 0.4	1.3 ± 0.4		1.1 ± 0.3	1.2 ± 0.3	1.1 ± 0.2	1.2 ± 0.3
CLung	1.0 ± 0.3	1.0 ± 0.4		0.9 ± 0.2	0.9 ± 0.3	0.9 ± 0.2	0.9 ± 0.2
Heart (Lt.)	3.4 ± 0.7	3.9 ± 1.1		3.0 ± 0.9	3.5 ± 1.2	3.2 ± 0.7	3.6 ± 1.0
Heart (Rt.)	2.3 ± 0.5	2.4 ± 0.6		1.9 ± 0.2	2.0 ± 0.2	2.0 ± 0.6	2.1 ± 0.6

**Table 3:** Absolute and relative differences in mean dose and BED between sequential boost and the simultaneous integrated boost using the same prescribed biologically effective dose with α/β = 10 Gy (BED_10_) and 3 Gy (BED_3_) for all structures of the ten studied patients (mean ± SD)

	Dose (Gy)	(SIB_10_/SEQ–1) × 100		Dose (Gy)	(SIB_3_/SEQ–1) × 100	
		Dose (%)	BED_10_ (%)		Dose (%)	BED_3_ (%)
PTV_boost_	−1.5 ± 0.7	−2 ± 1	0 ± 1	−3.0 ± 0.7	−5 ± 1	0 ± 2
PTV_breast_	−1.3 ± 0.8	−2 ± 1	−3 ± 1 ^a^	−0.6 ± 0.9	−1 ± 2	−2 ± 2 ^a^
ILung	−0.7 ± 0.4	−8 ± 4	−10 ± 4 ^a^	−0.8 ± 0.5	−9 ± 4	−11 ± 4 ^a^
CBreast	−0.1 ± 0.3	−6 ± 16	−6 ± 17	−0.1 ± 0.3	−5 ± 15	−6 ± 16
CLung	−0.1 ± 0.1	−7 ± 13	−8 ± 14	−0.1 ± 0.2	−6 ± 17	−7 ± 18
Heart_left_^b^	−0.3 ± 0.5	−10 ± 16	−12 ± 16	−0.2 ± 0.1	−6 ± 4	−8 ± 4 ^a^
Heart_right_^c^	−0.3 ± 0.4	−12 ± 17	−14 ± 19	−0.2 ± 0.6	−8 ± 24	−10 ± 25

^a^Indicates a significant difference (*p* <0.05) between the SEQ and SIB plans in term of BED

^b^Group of 6 patients with left-sided breast tumor

^c^Group of 4 patients with right-sided breast tumor

## Discussion

The present study used the biologically effective dose (BED) concept to compare the BED distribution between the breast sequential boost and simultaneously integrated boost schemes.

An iso-BED was calculated for the breast sequential boost (SEQ) prescribed dose giving in 2 Gy per fraction for 25 and 8 fractions for breast and boost target volumes respectively for each of α/β = 10 (BED_10_) and 3 (BED_3_) Gy. Based on the iso-effective prescribed dose of the sequential boost, the corresponding simultaneously integrated boost (SIB) prescribed doses were calculated. For each of ten breast patients, a SEQ IMRT plan and two SIB IMRT plans (one for each of BED_10_ (SIB_10_) and BED_3_ (SIB_3_)) were generated (Table 2). Corresponding 3D BED distributions were calculated. A comparison of the BED distributions and mean structures’ BED between the sequential and simultaneously integrated boost plans were performed.

The results showed that the SIB schemes are better than the SEQ schemes for PTV_breast_ (about 1 and 3 %), ipsi-lateral OARs (about 8 and 10 %) and contra-lateral OARs (about 6 and 7 %) in terms of dose and BED, respectively (Table 3). It is also can be seen from the smaller deviation of the mean values and the smaller SDs of the SIB targets BEDs compared to the SEQ targets BEDs that the targets prescribed BEDs are better achieved in SIB plans than in SEQ plans. Although the dose reductions are in agreement with previously reported results [3, 12], when using the BEDVH concept it becomes clear that biologically effective dose is considerably reduced. The SIB and SEQ plans have the same iso-BED, thus it is better to compare both plans based on the corresponding BEDs instead on doses. This is due to the difference between the fractionation that leads to different biological effects. Based on the BED comparisons, the SIB plans reduced the PTV_breast_ mean BED by about 3 % (Table 3), the ipsi-lateral lung and heart by about 10 %, and contra-lateral breast and lung by about 7 %. About 0.3 Gy cardiac dose reduction is reported in this study. One of the most current population-based analyses has estimated a linear increase in risk of major coronary events by 7.4 % for each increase of 1 Gy in the mean radiation dose delivered to the heart [23]. Therefore, we believe that the reported difference in cardiac dose is meaningful. This improvement is mainly due to the single step optimization of the SIB plan, compared to the SEQ planning comprising two separate steps for the breast and the boost plans. This allows the optimization algorithm to account for the dose from all fields in a one process and thus eliminate the breast hot-spot and reduce the OARs doses.

In this analysis, a simple and more traditional tangential field arrangement was used. Although other multi-field non-coplanar, IMRT or Volumetric Modulated Arc Therapy (VMAT) techniques would improve the conformality and dose homogeneity within the target volumes and may reduce OARs doses, our aim was to demonstrate superiority of the SIB approach as a matter of principle despite the use of *simplified* technique. Therefore, it is essential to compare techniques which differ only with respect to the planning algorithm, but not the irradiation angles. The influence of the irradiation technique is out of the scope of this study.

Recent clinical trials [1, 5, 6] were published proving the feasibility and well-tolerated toxicity of hypofractionation with SIB in early breast cancer. The comparison done here was based on the BED and hence it is important to consider the limitations of the LQ model and the BED calculations. The LQ model does not take into account the overall treatment time and potential volume effect. This limitation may be important when comparing treatment schemes differing on overall treatment time in terms of acute toxicity [10, 18]. Therefore, the assumption of overall treatment time independency may become inaccurate when comparing widely different overall treatment times such as in hypofractionated schemes [1, 5, 6, 24]. Generally, it is considered that the limitations of using the LQ model are mainly due to inaccuracies of accounting for repopulation, bi-fractionated treatments and high-dose fractions [25].

To account for variations due to uncertainty of α/β ratio, two different α/β ratios (10 and 3 Gy) were used for the tumor target volumes in the calculations of BED as generic values to account for a range of expected values. As the overall difference in the advantage for α/β = 10 Gy compared to 3 Gy is relatively low (Table 3), our results can be assumed representative also for other discussed values, e.g., α/β = 4 Gy [14, 15]. Note that the selection of two generic α/β ratios (10 Gy for target volumes and 3 Gy for OARs) is the reason to see BED hot-spots outside the target volumes (Fig. 1). This phenomenon appears when neighboring structures have different α/β ratios and results in a discontinuous BED distribution at the border of the structures. Clearly, Fig. 2 with an α/β ratio of 3 Gy for the breast tissue is more realistic.

## Conclusion

Biologically effective dose comparison between sequential and simultaneously integrated boost could be an important tool in plan evaluation and in understanding clinical consequences of unconventional dose schedules. It helped in demonstrating the advantages of the simultaneously integrated boost for breast cancer in terms of breast target volume and OARs doses.

## References

[1] Dellas K, Vonthein R, Zimmer J, Dinges S, Boicev AD, Andreas P (2014). Hypofractionation with simultaneous integrated boost for early breast cancer: results of the German multicenter phase II trial (ARO-2010-01). Strahlenther Onkol.

[2] Sedlmayer F, Sautter-Bihl ML, Budach W, Dunst J, Fastner G, Feyer P (2013). DEGRO practical guidelines: radiotherapy of breast cancer I : radiotherapy following breast conserving therapy for invasive breast cancer. Strahlenther Onkol.

[3] Alford SL, Prassas GN, Vogelesang CR, Leggett HJ, Hamilton CS (2013). Adjuvant breast radiotherapy using a simultaneous integrated boost: clinical and dosimetric perspectives. J Med Imaging Radiat Oncol.

[4] Bantema-Joppe EJ, Schilstra C, de Bock GH, Dolsma WV, Busz DM, Langendijk JA (2012). Simultaneous integrated boost irradiation after breast-conserving surgery: physician-rated toxicity and cosmetic outcome at 30 months’ follow-up. Int J Radiat Oncol Biol Phys.

[5] Scorsetti M, Alongi F, Fogliata A, Pentimalli S, Navarria P, Lobefalo F (2012). Phase I-II study of hypofractionated simultaneous integrated boost using volumetric modulated arc therapy for adjuvant radiation therapy in breast cancer patients: a report of feasibility and early toxicity results in the first 50 treatments. Radiat Oncol.

[6] Van Parijs H, Miedema G, Vinh-Hung V, Verbanck S, Adriaenssens N, Kerkhove D (2012). Short course radiotherapy with simultaneous integrated boost for stage I-II breast cancer, early toxicities of a randomized clinical trial. Radiat Oncol.

[7] Barendsen GW (1982). Dose fractionation, dose rate and iso-effect relationships for normal tissue responses. Int J Radiat Oncol Biol Phys.

[8] Fowler JF (1989). The linear-quadratic formula and progress in fractionated radiotherapy. Br J Radiol.

[9] Jones B, Dale RG, Deehan C, Hopkins KI, Morgan DAL (2001). The role of Biologically Effective Dose (BED) in clinical oncology. Clin Oncol (R Coll Radiol).

[10] Guerrero M, Li XA, Earl MA, Sarfaraz M, Kiggundu E (2004). Simultaneous integrated boost for breast cancer using IMRT: a radiobiological and treatment planning study. Int J Radiat Oncol Biol Phys.

[11] Gustafsson J, Nilsson P, Gleisner KS (2013). On the biologically effective dose (BED)-using convolution for calculating the effects of repair: I. Analytical considerations. Phys Med Biol.

[12] van der Laan HP, Dolsma WV, Maduro JH, Korevaar EW, Hollander M, Langendijk JA (2007). Three-dimensional conformal simultaneously integrated boost technique for breast-conserving radiotherapy. Int J Radiat Oncol Biol Phys.

[13] Sedlmayer F, Sautter-Bihl ML, Budach W, Dunst J, Feyer P, Fietkau R (2013). Is the simultaneously integrated boost (SIB) technique for early breast cancer ready to be adopted for routine adjuvant radiotherapy? Statement of the German and the Austrian Societies of Radiooncology (DEGRO/OGRO). Strahlenther Onkol.

[14] Haviland JS, Agrawal RK, Aird EG, Barrett A, Barrett-Lee PJ, Brown J, et al. The UK START (Standardisation of Breast Radiotherapy) Trials: 10-year follow-up results. In: Thirty-Fifth Annual CTRC-AACR San Antonio Breast Cancer Symposium; San Antonio, TX. Cancer Res; 2012: S4–1.

[15] Bentzen SM, Agrawal RK, Aird EG, Barrett JM, Barrett-Lee PJ, Bliss JM (2008). The UK Standardisation of Breast Radiotherapy (START) Trial A of radiotherapy hypofractionation for treatment of early breast cancer: a randomised trial. Lancet Oncol.

[16] Boersma LJ, van den Brink M, Bruce AM, Shouman T, Gras L, te Velde A (1998). Estimation of the incidence of late bladder and rectum complications after high-dose (70–78 GY) conformal radiotherapy for prostate cancer, using dose-volume histograms. Int J Radiat Oncol Biol Phys.

[17] Marks LB, Munley MT, Bentel GC, Zhou SM, Hollis D, Scarfone C (1997). Physical and biological predictors of changes in whole-lung function following thoracic irradiation. Int J Radiat Oncol Biol Phys.

[18] Wheldon TE, Deehan C, Wheldon EG, Barrett A (1998). The linear-quadratic transformation of dose-volume histograms in fractionated radiotherapy. Radiother Oncol.

[19] Lee SP, Leu MY, Smathers JB, McBride WH, Parker RG, Withers HR (1995). Biologically effective dose distribution based on the linear quadratic model and its clinical relevance. Int J Radiat Oncol Biol Phys.

[20] Jones LC, Hoban PW (2000). Treatment plan comparison using equivalent uniform biologically effective dose (EUBED). Phys Med Biol.

[21] Bartelink H, Horiot J-C, Poortmans P, Struikmans H, Van den Bogaert W, Barillot I (2001). Recurrence rates after treatment of breast cancer with standard radiotherapy with or without additional radiation. N Engl J Med.

[22] Deasy JO, Blanco AI, Clark VH (2003). CERR: a computational environment for radiotherapy research. Med Phys.

[23] Darby SC, Ewertz M, McGale P, Bennet AM, Blom-Goldman U, Brønnum D (2013). Risk of ischemic heart disease in women after radiotherapy for breast cancer. N Engl J Med.

[24] Chadha M, Vongtama D, Friedmann P, Parris C, Boolbol SK, Woode R (2012). Comparative acute toxicity from whole breast irradiation using 3-week accelerated schedule with concomitant boost and the 6.5-week conventional schedule with sequential boost for early-stage breast cancer. Clin Breast Cancer.

[25] Voyant C, Julian D, Roustit R, Biffi K, Lantieri C (2014). Biological effects and equivalent doses in radiotherapy: a software solution. Rep Pract Oncol Radiother.

